# Crystal structures of 3,5-bis­[(*E*)-3-hy­droxy­benzyl­idene]-1-methyl­piperidin-4-one and 3,5-bis­[(*E*)-2-chloro­benzyl­idene]-1-methyl­piperidin-4-one

**DOI:** 10.1107/S2056989015020976

**Published:** 2015-11-11

**Authors:** Yum Eryanti, Adel Zamri, Tati Herlina, Unang Supratman, Mohd Mustaqim Rosli, Hoong-Kun Fun

**Affiliations:** a Laboratory of Organic Synthesis, Department of Chemistry, Faculty of Mathematics and Natural Sciences, Riau University, Pekanbaru 26293, Indonesia; b Department of Chemistry, Faculty of Mathematics and Natural Sciences, Padjadjaran University, Jalan Raya Bandung-Sumedang Km 21, Jatinangor 45363, Sumedang, Indonesia; c Department of Chemistry, Faculty of Mathematics and Narural Sciences, Padjadjaran University, Jalan Raya Bandung-Sumedang Km 21, Jatinangor 45363, Sumedang, Indonesia; d X-ray Crystallography Unit, School of Physics, Universiti Sains Malaysia, 11800 USM, Penang, Malaysia; e Department of Pharmaceutical Chemistry, College of Pharmacy, King Saud University, Riyadh 11451, Saudi Arabia

**Keywords:** crystal structure, benzyl­idene, piperidinone, methyl­piperidin-4-one, hydrogen bonding

## Abstract

The title compounds, C_20_H_19_NO_3_, (1), and C_20_H_17_Cl_2_NO, (2), are the 3-hy­droxy­benzyl­idene and 2-chloro­benzyl­idene derivatives, respectively, of curcumin [systematic name: (1*E*,6*E*)-1,7-bis­(4-hy­droxy-3-meth­oxy­phen­yl)-1,6-hepta­diene-3,5-dione]. The dihedral angles between the benzene rings in each compound are 21.07 (6)° for (1) and 13.4 (3)° for (2). In both compounds, the piperidinone rings adopt a sofa confirmation and the methyl group attached to the N atom is in an equatorial position. In the crystal of (1), two pairs of O—H⋯N and O—H⋯O hydrogen bonds link the mol­ecules, forming chains along [10-1]. The chains are linked *via* C—H⋯O hydrogen bonds, forming undulating sheets parallel to the *ac* plane. In the crystal of (2), mol­ecules are linked by weak C—H⋯Cl hydrogen bonds, forming chains along the [204] direction. The chains are linked along the *a*-axis direction by π–π inter­actions [inter-centroid distance = 3.779 (4) Å]. For compound (2), the crystal studied was a non-merohedral twin with the refined ratio of the twin components being 0.116 (6):0.886 (6).

## Abstract 2

3,5-Bis[(*E*)-3-hy­droxy­benzyl­idene]-1-methyl­piperidin-4-one and the 2-chloro­benzyl­idene derivative are monocarbonyl analogues of curcumin, and the conformations of the two compounds are very similar. In the 3-hy­droxy­benzyl­idene compound, O—H⋯O, O—H⋯N and C—H⋯O hydrogen bonds link the mol­ecules, forming sheets lying parallel to the *ac* plane. In the crystal of the 2-chloro­benzyl­idene derivative, mol­ecules are linked *via* weak C—H⋯Cl hydrogen bonds, forming zigzag chains along the [204] direction.

## Chemical context   

Curcumin (diferuloyl­methane) is a naturally occurring biologically active compound, isolated from the root of the tumeric plant (*Curcuma longa*) (Dandia *et al.*, 2012 ▸). It has been shown to exhibit anti-oxidant (Rostom *et al.*, 2009 ▸), anti-inflammatory (Suzuki *et al.*, 2005 ▸), anti­viral (Kumar *et al.*, 2007 ▸) and anti­bacterial (Bandgar *et al.*, 2012 ▸) activities, and thus has potential against various malignant cancers, diabetes, allergies, arthritis and other chronic illnesses (Yadav *et al.*, 2010 ▸; Reddy *et al.*, 2009 ▸; Aggarwal *et al.*, 2003 ▸; Insuasty *et al.*, 2013 ▸; Wu *et al.*, 2013 ▸). For the purpose of finding new deriv­atives with increased systemic bioavailability and enhanced pharmacological activity (Zhao *et al.*, 2010 ▸), chemical modifications as well as the synthesis of curcumin analogues have been attempted by many research groups in order to find a better treatment for various diseases (Siddiqui *et al.*, 2006 ▸; Gregory *et al.*, 2013 ▸). Analogous compounds to (*E*)-3,5-bis­(benzyl­idene)-4-piperidones present noteworthy cytotoxic activity against leukemia cell lines and colon cancer, among others (Gregory *et al.*, 2013 ▸). Different substituents were designed to investigate and discuss the structure–activity relationship (Insuasty *et al.*, 2013 ▸). Herein, we report on the synthesis, characterization and crystal structures of two mono-carbonyl analogues of curcumin, namely *N*-methyl-(3*E*,5*E*)-3,5-bis­(3-hy­droxy­benzyl­idene)-4-piperidone (1) and *N*-methyl-(3*E*,5*E*)-3,5-bis­(2-chloro­benzyl­idene)-4-piperidone (2).
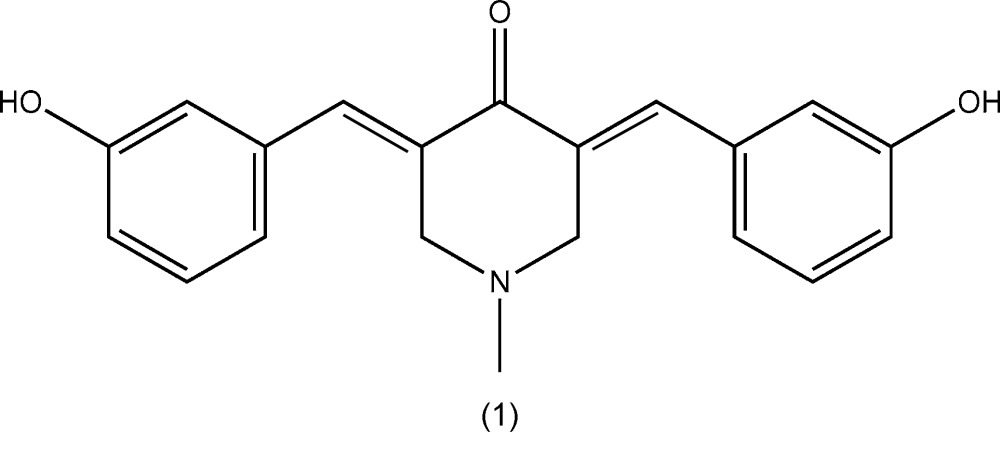
 
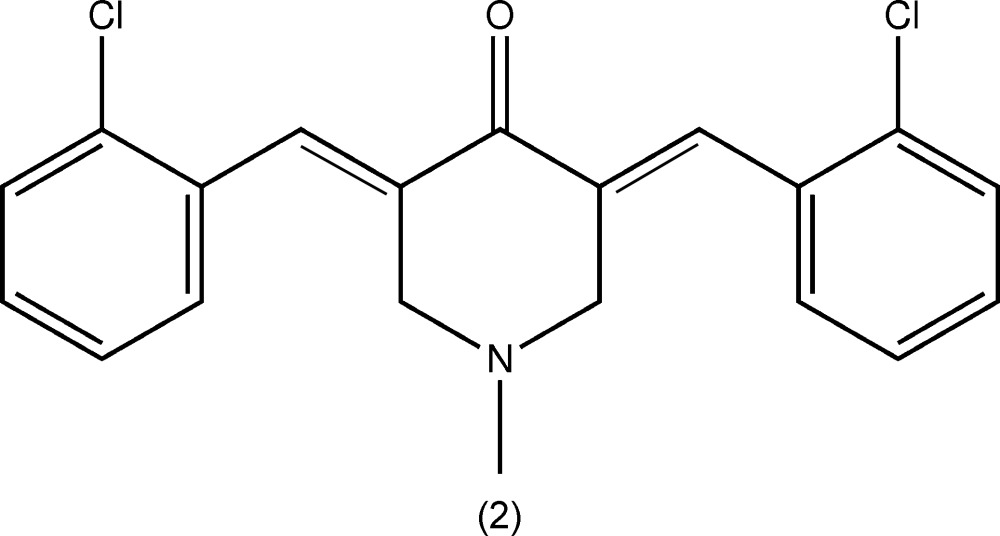


## Structural commentary   

**Figure 1:**
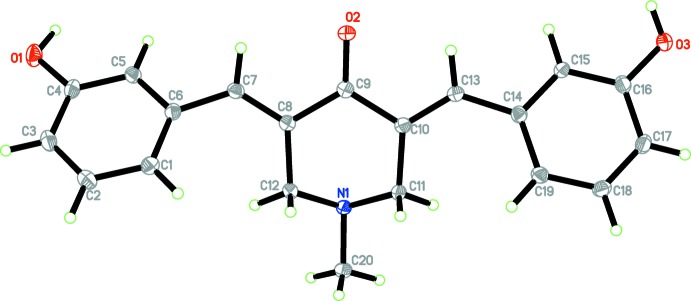
The mol­ecular structure of compound (1), showing 50% probability displacement ellipsoids and the atom labelling.

The mol­ecular structures of compounds (1) and (2) are shown in Figs. 1 ▸ and 2 ▸, respectively. Compound (1) crystallized in the triclinic space group *P* 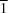 (*Z* = 2), while compound (2) crystallized in the monoclinic space group *P*2_1_/*n* (*Z* = 4).

The benzene rings (C1–C6 and C14–C19) are inclined to one another by 21.07 (6)° in (1) and by 13.4 (3)° in (2). Both compounds exhibit *E* conformations about the C7=C8 and C13=C10 bonds. In both compounds, the piperidinone ring (N1/C8–C12) adopts a sofa conformation with atom N1 displaced from the mean plane through the five C atoms (C8–C12) by 0.7052 (10) Å in (1) and 0.705 (5) Å in (2). The puckering parameters for the piperidinone ring conformation in (1) are *Q* = 0.5280 (12) Å, θ = 55.17 (14)° and φ = 353.08 (17)°, while for (2) they are *Q* = 0.526 (6) Å, θ = 126.1 (7)° and φ = 182.8 (8)°. In both compounds the methyl group attached to atom N1 is in an equatorial position on the piperidinone ring.

## Supra­molecular features   

**Figure 3:**
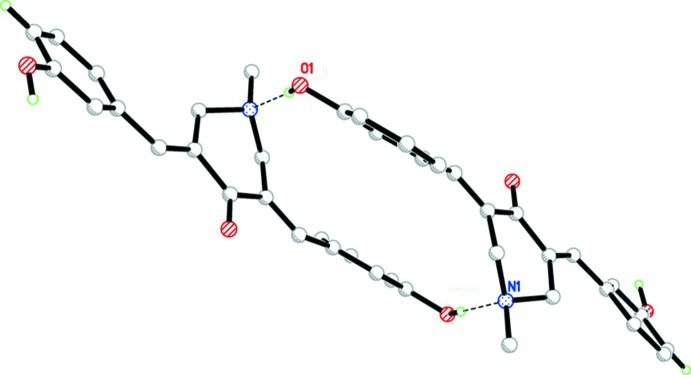
An inversion dimer found in compound (1), formed by O—H⋯N hydrogen bonds (dashed lines; see Table 1 ▸).

**Figure 4:**
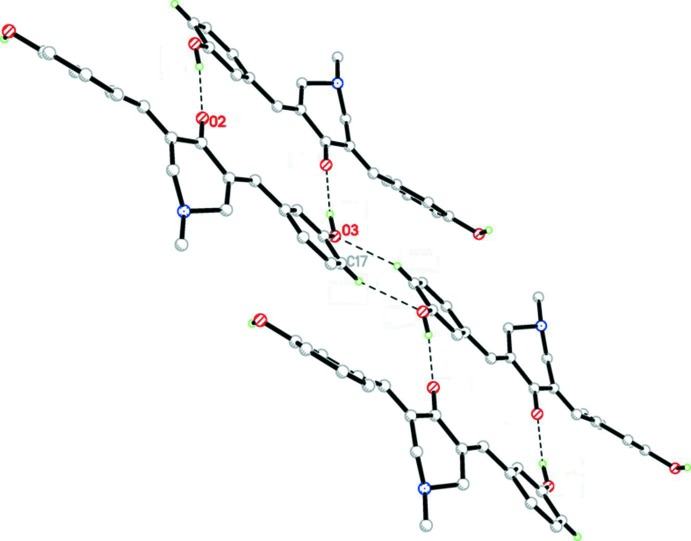
Inversion dimers found in compound (1), formed by O—H⋯O and C—H⋯N hydrogen bonds (dashed lines; see Table 1 ▸).

**Figure 5:**
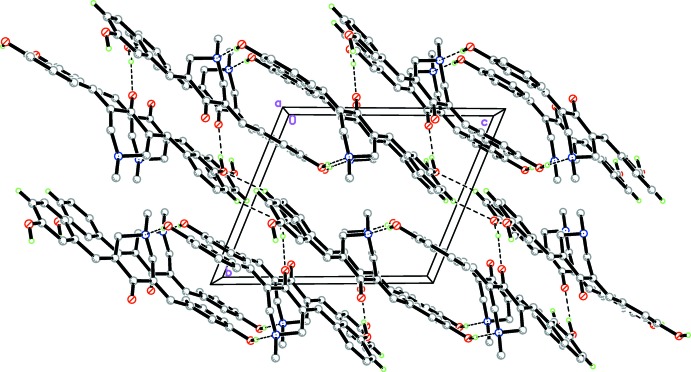
The crystal packing of compound (1), viewed along the *a* axis. Dashed lines indicate hydrogen bonds (see Table 1 ▸). H atoms not involved in the hydrogen bonding have been omitted for clarity.

In the crystal of compound (1), mol­ecules are linked *via* pairs of O—H⋯N hydrogen bonds, forming inversion dimers enclosing an 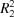
(18) ring motif (Table 1 ▸ and Fig. 3 ▸). These dimers are linked by pairs of O—H⋯O hydrogen bonds, enclosing an 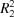
(18) ring motif, forming chains along [10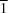] (Table 1 ▸ and Fig. 4 ▸). The chains are linked *via* pairs of C—H⋯O hydrogen bonds (Table 1 ▸ and Fig. 4 ▸), forming undulating sheets lying parallel to the *ac* plane (Fig. 5 ▸).

**Figure 6:**
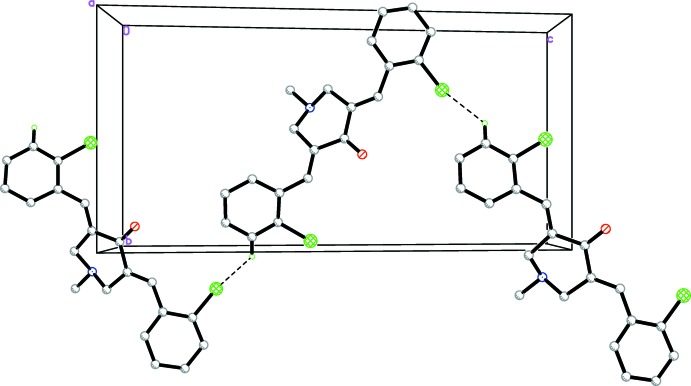
A view along the *a* axis of the crystal packing of compound (2), showing a zigzag chain formed by weak C—H⋯Cl hydrogen bonds (dashed lines; see Table 2 ▸). H atoms not involved in the hydrogen bonding have been omitted for clarity.

In the crystal of compound (2), mol­ecules are linked by a weak C4—H4*A*⋯Cl2^i^ hydrogen bond, forming zigzag chains along [204] (Table 2 ▸ and Fig. 6 ▸). The chains are linked along the *a-*axis direction by π–π inter­actions [*Cg*2⋯*Cg*3^i^ = 3.779 (4) Å, where *Cg*2 and *Cg*3 are the centroids of rings C1–C6 and C14–C19, respectively; symmetry code: (i) − *x* + 1, −*y*, −*z*].

## Database survey   

A search of the Cambridge Structural Database (CSD, Version 5.36, last update February 2015; Groom & Allen, 2014 ▸) of substructure (3*E*,5*E*)-3,5-di­benzyl­idene-1-methyl­piperidin-4-one gave 49 hits. One compound, 3,5-bis­(4-chloro­benzyl­idene)-1-methyl­piperidin-4-one, is the 4-chloro­benzyl­idene isomer of compound (2) (UNOXOL; Nesterov *et al.*, 2011 ▸). Here, the benzene rings are inclined to one another by 7.58 (8)°, compared to 21.07 (6)° in (1) and 13.4 (3)° in (2). The piperidinone ring also adopts a sofa conformation with the N atom displaced from the mean plane of the five C atoms by 0.7714 (15) Å, compared to 0.7052 (10) Å in (1) and 0.705 (5) Å in (2).

## Synthesis and crystallization   

Both compounds were synthesized according to a partially modified procedure of a previous report (Gregory *et al.*, 2013 ▸).

**Compound (1):** The corresponding *N*-methyl-4-piperidone (0.99 g, 0.01 mol), 3-hy­droxy­benzaldehyde (2.23 g, 0.02 mol), 40% aq. NaOH (0.7 ml) and 95% EtOH (5 ml) were mixed with stirring at room temperature for 30 min. The reaction mixture was subjected to microwave irradiation for 3 min at a power of 180 W and temperature of 333 K. The reaction product was cooled and cold water was added. The precipitate formed was filtered and recrystallized from a mixture of *n*-hexa­ne–ethyl acetate to afford dark yellowish crystals of compound (1) (yield: 3.4 g, 34.5%; m.p. 409–410 K). *R_f_* = 0.43 (*n*-hexa­ne:EtOAc = 1:1). UV (MeOH) λ_max_: 364 nm (∊ 4,600). IR (KBr) ν_max_ cm^−1^: 3400, 1658, 1600 and 1504 cm^−1^. ^1^H NMR (500 MHz, CDCl3): δ (p.p.m.) 8.04 (2H, *s*), 7.31 (2H, *d*, *J* = 7.5 Hz), 7.26 (2H, *t*, *J* = 7.5 Hz), 6.99 (2H, *d*, *J* = 8.0 Hz), 6.93 (2H, *t*, *J* = 7.5 Hz), 3.72 (4H, *s*) and 2.41 (3H, *s*). ^13^C NMR (125 MHz, CDCl3): δ (p.p.m.) 185.9, 156.6, 133.2, 130.7, 130.5, 130.3, 122.6, 119.4, 115.7, 57.2, 45.2. HR–ESI–TOFMS: calculated for C_20_H_19_NO_3_ [*M* + H]^+^, *m*/*z* 321.1365, found *m*/*z* 322.1434.

**Compound (2):** The corresponding *N*-methyl-4-piperidone (0.98 g, 0.01 mol), 2-chloro­benzaldehyde (2.20 g, 0.02 mol), 40% aq. NaOH (0.7 ml) and 95% EtOH (5 ml) was stirred at room temperature for 30 min. The reaction mixture was subjected to microwave irradiation for 3 min at a power of 180 W and temperature of 333 K. The reaction product was cooled and cold water was added. The precipitate formed was filtered and recrystallized from a mixture of *n*-hexa­ne–ethyl acetate to afford yellowish crystals of compound (2) (yield: 3.8 g, 38.4%; m.p. 408–410 K). *R_f_* = 0.60 (CH_2_Cl_2_:MeOH = 9.5:0.5). UV (MeOH) λ_max_: 309 nm (∊ 4,400). IR (KBr) ν_max_ cm^−1^: 3328, 1640 cm^−1^. ^1^H NMR (500 MHz, CDCl3): δ (p.p.m.) 8.00 (2H, *s*), 7.46 (2H, *dd*, *J* = 8.0, 1.5 Hz), 7.31 (2H, *dd*, *J* = 8.0, 1.5 Hz), 7.30 (2H, *d*, *J* = 7.5 Hz), 7.24 (2H, *dd*, *J* = 7.5, 1.5 Hz), 3.61 (4H, *s*), 2.37 (3H, *s*). ^13^C NMR (125 MHz, CDCl3): δ (p.p.m.) 186.1, 135.2, 134.3, 134.0, 133.6, 130.3, 130.0, 129.9, 126.4, 56.7, 45.5. HR–ESI–TOFMS: calculated for C_20_H_17_Cl_2_NO [*M* + H]^+^, *m*/*z* 357.0687, found *m*/*z* 358.0776.

## Refinement   

Crystal data, data collection and structure refinement details are summarized in Table 3 ▸. The O-bound H atoms were located in difference Fourier maps and freely refined. The remaining H atoms were positioned geometrically and refined using a riding model: C—H = 0.95–0.99 Å with *U*_iso_(H) = 1.5*U*_eq_(C-meth­yl) and 1.2*U*_eq_(C) for other H atoms. A rotating group model was applied to the methyl groups. For compound (2) the crystal studied was a non-merohedral twin with a ratio of the twin components of 0.116 (6):0.886 (6).

## Supplementary Material

Crystal structure: contains datablock(s) 1, 2, global. DOI: 10.1107/S2056989015020976/su5232sup1.cif

Structure factors: contains datablock(s) 1. DOI: 10.1107/S2056989015020976/su52321sup4.hkl

Structure factors: contains datablock(s) 2. DOI: 10.1107/S2056989015020976/su52322sup5.hkl

Supporting information file. DOI: 10.1107/S2056989015020976/su52321sup4.cml

Supporting information file. DOI: 10.1107/S2056989015020976/su52322sup5.cml

CCDC references: 1435229, 1052718

Additional supporting information:  crystallographic information; 3D view; checkCIF report

## Figures and Tables

**Figure 2:**
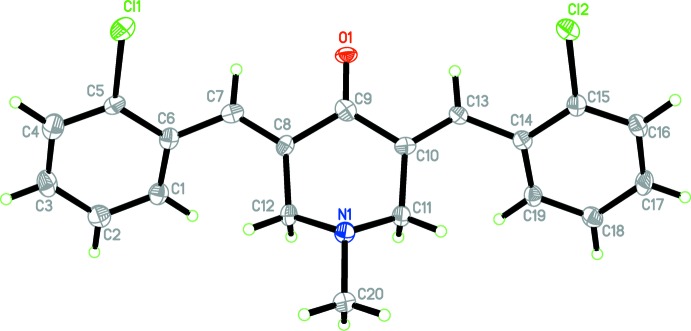
The mol­ecular structure of compound (2), showing 50% probability displacement ellipsoids and the atom labelling.

**Table 1:** Hydrogen-bond geometry (Å, °) for (1)

*D*—H⋯*A*	*D*—H	H⋯*A*	*D*⋯*A*	*D*—H⋯*A*
O1—H1*O*1⋯N1^i^	0.96 (2)	1.81 (2)	2.7278 (14)	160 (2)
O3—H1*O*3⋯O2^ii^	0.88 (2)	1.87 (2)	2.7359 (15)	171 (2)
C17—H17*A*⋯O3^iii^	0.95	2.51	3.4032 (16)	157

Symmetry codes: (i) 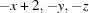; (ii) 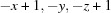; (iii) 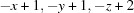.

**Table 2:** Hydrogen-bond geometry (Å, °) for (2)

*D*—H⋯*A*	*D*—H	H⋯*A*	*D*⋯*A*	*D*—H⋯*A*
C4—H4*A*⋯Cl2^i^	0.95	2.85	3.587 (7)	135

Symmetry code: (i) 
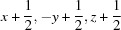
.

**Table 3:** Experimental details

	(1)	(2)
Crystal data		
Chemical formula	C_20_H_19_NO_3_	C_20_H_17_Cl_2_NO
*M*_r_	321.36	358.24
Crystal system, space group	Triclinic, *P* 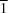	Monoclinic, *P*2_1_/*n*
Temperature (K)	100	100
*a*, *b*, *c* (Å)	7.4852 (6), 9.8588 (9), 11.6115 (10)	7.540 (3), 10.623 (4), 21.119 (7)
α, β, γ (°)	111.7924 (17), 96.7983 (18), 92.8848 (17)	90, 98.671 (5), 90
*V* (Å^3^)	785.90 (12)	1672.2 (10)
*Z*	2	4
Radiation type	Mo *K*α	Mo *K*α
μ (mm^−1^)	0.09	0.39
Crystal size (mm)	0.29 × 0.24 × 0.11	0.32 × 0.08 × 0.08

Data collection		
Diffractometer	Bruker APEX DUO CCD area detector	Bruker APEX DUO CCD area detector
Absorption correction	Multi-scan (*SADABS*; Bruker, 2009 ▸)	Multi-scan (*SADABS*; Bruker, 2009 ▸)
No. of measured, independent and observed [*I* > 2σ(*I*)] reflections	10462, 3562, 3133	3105, 3105, 2591
*R*_int_	0.021	0.084
(sin θ/λ)_max_ (Å^−1^)	0.650	0.606

Refinement		
*R*[*F* ^2^ > 2σ(*F* ^2^)], *wR*(*F* ^2^), *S*	0.039, 0.117, 1.04	0.077, 0.192, 1.18
No. of reflections	3562	3105
No. of parameters	226	218
H-atom treatment	H atoms treated by a mixture of independent and constrained refinement	H-atom parameters constrained
Δρ_max_, Δρ_min_ (e Å^−3^)	0.37, −0.21	0.45, −0.43

Computer programs: *APEX2* and *SAINT* (Bruker, 2009 ▸), *SHELXS* and *SHELXTL* (Sheldrick, 2008 ▸), *SHELXL2014* (Sheldrick, 2015 ▸), *PLATON* (Spek, 2009 ▸) and *publCIF* (Westrip, 2010 ▸).

## supplementary crystallographic information

### (1) 3,5-Bis[(E)-3-hydroxybenzylidene]-1-methylpiperidin-4-one Crystal data

**Table:**

C_20_H_19_NO_3_	*Z* = 2
*M**_r_* = 321.36	*F*(000) = 340
Triclinic, *P*1	*D*_x_ = 1.358 Mg m^−^^3^
*a* = 7.4852 (6) Å	Mo *K*α radiation, λ = 0.71073 Å
*b* = 9.8588 (9) Å	Cell parameters from 3138 reflections
*c* = 11.6115 (10) Å	θ = 2.8–32.1°
α = 111.7924 (17)°	µ = 0.09 mm^−^^1^
β = 96.7983 (18)°	*T* = 100 K
γ = 92.8848 (17)°	Block, orange
*V* = 785.90 (12) Å^3^	0.29 × 0.24 × 0.11 mm

### (1) 3,5-Bis[(E)-3-hydroxybenzylidene]-1-methylpiperidin-4-one Data collection

**Table:**

Bruker APEX DUO CCD area-detector diffractometer	3133 reflections with *I* > 2σ(*I*)
Radiation source: fine-focus sealed tube	*R*_int_ = 0.021
φ and ω scans	θ_max_ = 27.5°, θ_min_ = 1.9°
Absorption correction: multi-scan (*SADABS*; Bruker, 2009)	*h* = −9→9
	*k* = −12→12
10462 measured reflections	*l* = −15→15
3562 independent reflections	

### (1) 3,5-Bis[(E)-3-hydroxybenzylidene]-1-methylpiperidin-4-one Refinement

**Table:**

Refinement on *F*^2^	0 restraints
Least-squares matrix: full	Hydrogen site location: mixed
*R*[*F*^2^ > 2σ(*F*^2^)] = 0.039	H atoms treated by a mixture of independent and constrained refinement
*wR*(*F*^2^) = 0.117	*w* = 1/[σ^2^(*F*_o_^2^) + (0.0694*P*)^2^ + 0.2741*P*] where *P* = (*F*_o_^2^ + 2*F*_c_^2^)/3
*S* = 1.04	(Δ/σ)_max_ < 0.001
3562 reflections	Δρ_max_ = 0.37 e Å^−^^3^
226 parameters	Δρ_min_ = −0.21 e Å^−^^3^

### (1) 3,5-Bis[(E)-3-hydroxybenzylidene]-1-methylpiperidin-4-one Special details

**Table:**

Experimental. The crystal was placed in the cold stream of an Oxford Cryosystems Cobra open-flow nitrogen cryostat (Cosier & Glazer, 1986) operating at 100.0 (1) K.
Geometry. All e.s.d.'s (except the e.s.d. in the dihedral angle between two l.s. planes) are estimated using the full covariance matrix. The cell e.s.d.'s are taken into account individually in the estimation of e.s.d.'s in distances, angles and torsion angles; correlations between e.s.d.'s in cell parameters are only used when they are defined by crystal symmetry. An approximate (isotropic) treatment of cell e.s.d.'s is used for estimating e.s.d.'s involving l.s. planes.

### (1) 3,5-Bis[(E)-3-hydroxybenzylidene]-1-methylpiperidin-4-one Fractional atomic coordinates and isotropic or equivalent isotropic displacement parameters (Å^2^)

**Table:**

	*x*	*y*	*z*	*U*_iso_*/*U*_eq_	
O1	1.16705 (13)	−0.33469 (10)	−0.27862 (8)	0.0217 (2)	
O2	0.78550 (12)	−0.06971 (10)	0.30379 (9)	0.0219 (2)	
O3	0.36575 (12)	0.35383 (10)	0.81746 (9)	0.0197 (2)	
N1	1.15110 (13)	0.28041 (10)	0.38630 (9)	0.0139 (2)	
C1	1.36371 (16)	−0.10777 (13)	0.09923 (11)	0.0159 (2)	
H1A	1.4117	−0.0550	0.1849	0.019*	
C2	1.47889 (17)	−0.16263 (13)	0.01131 (12)	0.0182 (3)	
H2A	1.6059	−0.1478	0.0376	0.022*	
C3	1.41151 (17)	−0.23866 (13)	−0.11397 (12)	0.0184 (3)	
H3A	1.4922	−0.2770	−0.1728	0.022*	
C4	1.22557 (17)	−0.25933 (13)	−0.15432 (11)	0.0159 (3)	
C5	1.10880 (16)	−0.20577 (12)	−0.06675 (11)	0.0145 (2)	
H5A	0.9819	−0.2202	−0.0936	0.017*	
C6	1.17643 (16)	−0.13047 (12)	0.06113 (11)	0.0137 (2)	
C7	1.04624 (16)	−0.09053 (12)	0.14976 (11)	0.0141 (2)	
H7A	0.9348	−0.1514	0.1238	0.017*	
C8	1.06141 (16)	0.02010 (12)	0.26281 (11)	0.0136 (2)	
C9	0.91333 (16)	0.02744 (13)	0.33844 (11)	0.0152 (2)	
C10	0.92406 (16)	0.15518 (12)	0.45975 (11)	0.0139 (2)	
C11	1.08023 (16)	0.27250 (12)	0.49664 (11)	0.0144 (2)	
H11A	1.0401	0.3687	0.5454	0.017*	
H11B	1.1779	0.2515	0.5511	0.017*	
C12	1.21650 (16)	0.13979 (12)	0.31593 (11)	0.0148 (2)	
H12A	1.3070	0.1144	0.3721	0.018*	
H12B	1.2760	0.1484	0.2468	0.018*	
C13	0.78975 (16)	0.15721 (12)	0.52792 (11)	0.0145 (2)	
H13A	0.7002	0.0755	0.4914	0.017*	
C14	0.75909 (16)	0.26419 (12)	0.64853 (11)	0.0136 (2)	
C15	0.58209 (16)	0.25978 (12)	0.67642 (11)	0.0143 (2)	
H15A	0.4913	0.1888	0.6179	0.017*	
C16	0.53717 (16)	0.35752 (12)	0.78829 (11)	0.0150 (2)	
C17	0.66948 (17)	0.46108 (13)	0.87504 (11)	0.0173 (3)	
H17A	0.6396	0.5296	0.9509	0.021*	
C18	0.84563 (17)	0.46288 (13)	0.84920 (11)	0.0165 (3)	
H18A	0.9366	0.5320	0.9093	0.020*	
C19	0.89262 (16)	0.36657 (13)	0.73795 (11)	0.0154 (2)	
H19A	1.0142	0.3699	0.7224	0.018*	
C20	1.29894 (17)	0.39929 (13)	0.42690 (12)	0.0183 (3)	
H20A	1.2574	0.4907	0.4814	0.027*	
H20B	1.3364	0.4123	0.3533	0.027*	
H20C	1.4017	0.3742	0.4731	0.027*	
H1O1	1.044 (3)	−0.321 (2)	−0.302 (2)	0.049 (6)*	
H1O3	0.311 (3)	0.266 (2)	0.7732 (18)	0.038 (5)*	

### (1) 3,5-Bis[(E)-3-hydroxybenzylidene]-1-methylpiperidin-4-one Atomic displacement parameters (Å^2^)

**Table:**

	*U*^11^	*U*^22^	*U*^33^	*U*^12^	*U*^13^	*U*^23^
O1	0.0232 (5)	0.0259 (5)	0.0132 (4)	0.0063 (4)	0.0045 (4)	0.0032 (4)
O2	0.0192 (5)	0.0202 (4)	0.0187 (5)	−0.0078 (3)	0.0065 (4)	−0.0011 (4)
O3	0.0171 (4)	0.0197 (4)	0.0178 (5)	−0.0004 (3)	0.0076 (3)	0.0008 (4)
N1	0.0144 (5)	0.0132 (4)	0.0126 (5)	−0.0025 (4)	0.0037 (4)	0.0030 (4)
C1	0.0172 (6)	0.0146 (5)	0.0154 (6)	0.0012 (4)	0.0019 (4)	0.0055 (4)
C2	0.0142 (6)	0.0200 (6)	0.0225 (6)	0.0028 (4)	0.0044 (5)	0.0100 (5)
C3	0.0185 (6)	0.0192 (6)	0.0198 (6)	0.0053 (5)	0.0085 (5)	0.0079 (5)
C4	0.0210 (6)	0.0139 (5)	0.0132 (6)	0.0029 (4)	0.0044 (5)	0.0047 (4)
C5	0.0142 (5)	0.0140 (5)	0.0157 (6)	0.0014 (4)	0.0035 (4)	0.0058 (4)
C6	0.0158 (6)	0.0109 (5)	0.0151 (6)	0.0014 (4)	0.0044 (4)	0.0053 (4)
C7	0.0136 (5)	0.0147 (5)	0.0141 (6)	−0.0003 (4)	0.0021 (4)	0.0058 (4)
C8	0.0129 (5)	0.0137 (5)	0.0142 (6)	0.0008 (4)	0.0025 (4)	0.0053 (4)
C9	0.0147 (6)	0.0152 (5)	0.0151 (6)	0.0001 (4)	0.0029 (4)	0.0049 (4)
C10	0.0138 (5)	0.0141 (5)	0.0126 (5)	−0.0005 (4)	0.0015 (4)	0.0042 (4)
C11	0.0148 (6)	0.0151 (5)	0.0116 (5)	−0.0017 (4)	0.0029 (4)	0.0031 (4)
C12	0.0137 (5)	0.0148 (5)	0.0147 (6)	−0.0007 (4)	0.0043 (4)	0.0037 (4)
C13	0.0142 (5)	0.0140 (5)	0.0137 (6)	−0.0012 (4)	0.0014 (4)	0.0039 (4)
C14	0.0155 (6)	0.0136 (5)	0.0124 (5)	0.0009 (4)	0.0029 (4)	0.0056 (4)
C15	0.0153 (6)	0.0142 (5)	0.0118 (5)	−0.0013 (4)	0.0019 (4)	0.0036 (4)
C16	0.0153 (6)	0.0151 (5)	0.0152 (6)	0.0017 (4)	0.0045 (4)	0.0058 (4)
C17	0.0230 (6)	0.0145 (5)	0.0130 (6)	0.0006 (5)	0.0044 (5)	0.0035 (4)
C18	0.0191 (6)	0.0149 (5)	0.0138 (6)	−0.0036 (4)	−0.0004 (4)	0.0052 (4)
C19	0.0146 (6)	0.0175 (5)	0.0146 (6)	−0.0008 (4)	0.0022 (4)	0.0070 (5)
C20	0.0183 (6)	0.0153 (5)	0.0188 (6)	−0.0044 (4)	0.0047 (5)	0.0039 (5)

### (1) 3,5-Bis[(E)-3-hydroxybenzylidene]-1-methylpiperidin-4-one Geometric parameters (Å, º)

**Table:**

O1—C4	1.3596 (15)	C9—C10	1.4908 (16)
O1—H1O1	0.96 (2)	C10—C13	1.3476 (16)
O2—C9	1.2351 (14)	C10—C11	1.5051 (15)
O3—C16	1.3674 (14)	C11—H11A	0.9900
O3—H1O3	0.87 (2)	C11—H11B	0.9900
N1—C12	1.4668 (15)	C12—H12A	0.9900
N1—C20	1.4684 (14)	C12—H12B	0.9900
N1—C11	1.4697 (15)	C13—C14	1.4595 (16)
C1—C2	1.3868 (17)	C13—H13A	0.9500
C1—C6	1.3996 (17)	C14—C15	1.4030 (16)
C1—H1A	0.9500	C14—C19	1.4038 (16)
C2—C3	1.3824 (18)	C15—C16	1.3912 (16)
C2—H2A	0.9500	C15—H15A	0.9500
C3—C4	1.3944 (18)	C16—C17	1.3915 (17)
C3—H3A	0.9500	C17—C18	1.3871 (17)
C4—C5	1.3910 (16)	C17—H17A	0.9500
C5—C6	1.4064 (16)	C18—C19	1.3872 (17)
C5—H5A	0.9500	C18—H18A	0.9500
C6—C7	1.4625 (16)	C19—H19A	0.9500
C7—C8	1.3472 (16)	C20—H20A	0.9800
C7—H7A	0.9500	C20—H20B	0.9800
C8—C9	1.4819 (16)	C20—H20C	0.9800
C8—C12	1.5076 (15)		
			
C4—O1—H1O1	112.0 (13)	C10—C11—H11A	109.3
C16—O3—H1O3	108.1 (12)	N1—C11—H11B	109.3
C12—N1—C20	110.15 (9)	C10—C11—H11B	109.3
C12—N1—C11	109.61 (9)	H11A—C11—H11B	108.0
C20—N1—C11	109.52 (9)	N1—C12—C8	110.31 (9)
C2—C1—C6	119.72 (11)	N1—C12—H12A	109.6
C2—C1—H1A	120.1	C8—C12—H12A	109.6
C6—C1—H1A	120.1	N1—C12—H12B	109.6
C3—C2—C1	120.94 (11)	C8—C12—H12B	109.6
C3—C2—H2A	119.5	H12A—C12—H12B	108.1
C1—C2—H2A	119.5	C10—C13—C14	130.67 (11)
C2—C3—C4	120.23 (11)	C10—C13—H13A	114.7
C2—C3—H3A	119.9	C14—C13—H13A	114.7
C4—C3—H3A	119.9	C15—C14—C19	118.59 (11)
O1—C4—C5	123.05 (11)	C15—C14—C13	116.26 (10)
O1—C4—C3	117.67 (11)	C19—C14—C13	125.13 (11)
C5—C4—C3	119.27 (11)	C16—C15—C14	121.15 (11)
C4—C5—C6	120.74 (11)	C16—C15—H15A	119.4
C4—C5—H5A	119.6	C14—C15—H15A	119.4
C6—C5—H5A	119.6	O3—C16—C15	121.89 (11)
C1—C6—C5	119.07 (11)	O3—C16—C17	118.25 (11)
C1—C6—C7	122.82 (11)	C15—C16—C17	119.85 (11)
C5—C6—C7	117.90 (10)	C18—C17—C16	119.08 (11)
C8—C7—C6	129.38 (11)	C18—C17—H17A	120.5
C8—C7—H7A	115.3	C16—C17—H17A	120.5
C6—C7—H7A	115.3	C17—C18—C19	121.80 (11)
C7—C8—C9	117.96 (10)	C17—C18—H18A	119.1
C7—C8—C12	124.30 (10)	C19—C18—H18A	119.1
C9—C8—C12	117.72 (10)	C18—C19—C14	119.49 (11)
O2—C9—C8	121.55 (11)	C18—C19—H19A	120.3
O2—C9—C10	120.44 (11)	C14—C19—H19A	120.3
C8—C9—C10	118.00 (10)	N1—C20—H20A	109.5
C13—C10—C9	116.70 (10)	N1—C20—H20B	109.5
C13—C10—C11	124.79 (10)	H20A—C20—H20B	109.5
C9—C10—C11	118.51 (10)	N1—C20—H20C	109.5
N1—C11—C10	111.60 (9)	H20A—C20—H20C	109.5
N1—C11—H11A	109.3	H20B—C20—H20C	109.5
			
C6—C1—C2—C3	0.52 (18)	C12—N1—C11—C10	−60.87 (12)
C1—C2—C3—C4	1.06 (18)	C20—N1—C11—C10	178.18 (10)
C2—C3—C4—O1	179.88 (11)	C13—C10—C11—N1	−152.54 (12)
C2—C3—C4—C5	−1.56 (18)	C9—C10—C11—N1	26.59 (15)
O1—C4—C5—C6	178.98 (11)	C20—N1—C12—C8	−174.22 (9)
C3—C4—C5—C6	0.50 (17)	C11—N1—C12—C8	65.22 (12)
C2—C1—C6—C5	−1.56 (17)	C7—C8—C12—N1	142.85 (12)
C2—C1—C6—C7	172.99 (11)	C9—C8—C12—N1	−35.19 (14)
C4—C5—C6—C1	1.06 (17)	C9—C10—C13—C14	−179.80 (12)
C4—C5—C6—C7	−173.76 (10)	C11—C10—C13—C14	−0.7 (2)
C1—C6—C7—C8	31.69 (19)	C10—C13—C14—C15	160.79 (13)
C5—C6—C7—C8	−153.70 (12)	C10—C13—C14—C19	−20.9 (2)
C6—C7—C8—C9	−174.71 (11)	C19—C14—C15—C16	2.09 (18)
C6—C7—C8—C12	7.3 (2)	C13—C14—C15—C16	−179.43 (11)
C7—C8—C9—O2	4.94 (18)	C14—C15—C16—O3	−179.37 (11)
C12—C8—C9—O2	−176.91 (11)	C14—C15—C16—C17	−0.52 (18)
C7—C8—C9—C10	−176.05 (11)	O3—C16—C17—C18	177.59 (11)
C12—C8—C9—C10	2.11 (16)	C15—C16—C17—C18	−1.30 (18)
O2—C9—C10—C13	0.53 (18)	C16—C17—C18—C19	1.55 (19)
C8—C9—C10—C13	−178.49 (11)	C17—C18—C19—C14	0.05 (18)
O2—C9—C10—C11	−178.66 (11)	C15—C14—C19—C18	−1.85 (17)
C8—C9—C10—C11	2.31 (17)	C13—C14—C19—C18	179.83 (11)

### (1) 3,5-Bis[(E)-3-hydroxybenzylidene]-1-methylpiperidin-4-one Hydrogen-bond geometry (Å, º)

**Table:**

*D*—H···*A*	*D*—H	H···*A*	*D*···*A*	*D*—H···*A*
O1—H1*O*1···N1^i^	0.96 (2)	1.81 (2)	2.7278 (14)	160 (2)
O3—H1*O*3···O2^ii^	0.88 (2)	1.87 (2)	2.7359 (15)	171 (2)
C17—H17*A*···O3^iii^	0.95	2.51	3.4032 (16)	157

Symmetry codes: (i) −*x*+2, −*y*, −*z*; (ii) −*x*+1, −*y*, −*z*+1; (iii) −*x*+1, −*y*+1, −*z*+2.

### (2) 3,5-Bis[(E)-2-chlorobenzylidene]-1-methylpiperidin-4-one Crystal data

**Table:**

C_20_H_17_Cl_2_NO	*F*(000) = 744
*M**_r_* = 358.24	*D*_x_ = 1.423 Mg m^−^^3^
Monoclinic, *P*2_1_/*n*	Mo *K*α radiation, λ = 0.71073 Å
*a* = 7.540 (3) Å	Cell parameters from 3908 reflections
*b* = 10.623 (4) Å	θ = 2.7–29.2°
*c* = 21.119 (7) Å	µ = 0.39 mm^−^^1^
β = 98.671 (5)°	*T* = 100 K
*V* = 1672.2 (10) Å^3^	Needle, yellow
*Z* = 4	0.32 × 0.08 × 0.08 mm

### (2) 3,5-Bis[(E)-2-chlorobenzylidene]-1-methylpiperidin-4-one Data collection

**Table:**

Bruker APEX DUO CCD area-detector diffractometer	2591 reflections with *I* > 2σ(*I*)
Radiation source: fine-focus sealed tube	*R*_int_ = 0.084
φ and ω scans	θ_max_ = 25.5°, θ_min_ = 2.0°
Absorption correction: multi-scan (*SADABS*; Bruker, 2009)	*h* = −9→9
	*k* = −12→12
3105 measured reflections	*l* = −5→25
3105 independent reflections	

### (2) 3,5-Bis[(E)-2-chlorobenzylidene]-1-methylpiperidin-4-one Refinement

**Table:**

Refinement on *F*^2^	Primary atom site location: structure-invariant direct methods
Least-squares matrix: full	Secondary atom site location: difference Fourier map
*R*[*F*^2^ > 2σ(*F*^2^)] = 0.077	Hydrogen site location: inferred from neighbouring sites
*wR*(*F*^2^) = 0.192	H-atom parameters constrained
*S* = 1.18	*w* = 1/[σ^2^(*F*_o_^2^) + 13.4429*P*] where *P* = (*F*_o_^2^ + 2*F*_c_^2^)/3
3105 reflections	(Δ/σ)_max_ < 0.001
218 parameters	Δρ_max_ = 0.45 e Å^−^^3^
0 restraints	Δρ_min_ = −0.43 e Å^−^^3^

### (2) 3,5-Bis[(E)-2-chlorobenzylidene]-1-methylpiperidin-4-one Special details

**Table:**

Experimental. The crystal was placed in the cold stream of an Oxford Cryosystems Cobra open-flow nitrogen cryostat (Cosier & Glazer, 1986) operating at 100.0 (1) K.
Geometry. All e.s.d.'s (except the e.s.d. in the dihedral angle between two l.s. planes) are estimated using the full covariance matrix. The cell e.s.d.'s are taken into account individually in the estimation of e.s.d.'s in distances, angles and torsion angles; correlations between e.s.d.'s in cell parameters are only used when they are defined by crystal symmetry. An approximate (isotropic) treatment of cell e.s.d.'s is used for estimating e.s.d.'s involving l.s. planes.
Refinement. Refined as a 2-component twin.

### (2) 3,5-Bis[(E)-2-chlorobenzylidene]-1-methylpiperidin-4-one Fractional atomic coordinates and isotropic or equivalent isotropic displacement parameters (Å^2^)

**Table:**

	*x*	*y*	*z*	*U*_iso_*/*U*_eq_	
Cl1	0.8841 (2)	0.46525 (14)	0.06491 (7)	0.0261 (4)	
Cl2	0.6925 (2)	−0.18217 (14)	−0.22186 (7)	0.0242 (4)	
O1	0.6046 (5)	0.0962 (4)	−0.04698 (18)	0.0193 (9)	
N1	0.9749 (6)	−0.1131 (4)	0.0667 (2)	0.0179 (10)	
C1	0.7905 (8)	0.1999 (6)	0.1897 (3)	0.0205 (13)	
H1A	0.7492	0.1153	0.1892	0.025*	
C2	0.8325 (8)	0.2615 (6)	0.2487 (3)	0.0244 (13)	
H2A	0.8193	0.2199	0.2876	0.029*	
C3	0.8940 (8)	0.3854 (6)	0.2491 (3)	0.0263 (14)	
H3A	0.9266	0.4275	0.2889	0.032*	
C4	0.9085 (8)	0.4479 (6)	0.1928 (3)	0.0264 (14)	
H4A	0.9477	0.5329	0.1934	0.032*	
C5	0.8648 (8)	0.3841 (5)	0.1355 (3)	0.0182 (12)	
C6	0.8072 (7)	0.2581 (5)	0.1317 (3)	0.0178 (12)	
C7	0.7554 (7)	0.1956 (5)	0.0699 (3)	0.0184 (12)	
H7A	0.6965	0.2461	0.0359	0.022*	
C8	0.7825 (7)	0.0744 (5)	0.0562 (3)	0.0156 (12)	
C9	0.7007 (7)	0.0275 (6)	−0.0089 (3)	0.0181 (12)	
C10	0.7323 (7)	−0.1071 (5)	−0.0242 (3)	0.0158 (12)	
C11	0.8378 (8)	−0.1877 (5)	0.0269 (3)	0.0186 (12)	
H11A	0.7555	−0.2250	0.0542	0.022*	
H11B	0.8959	−0.2573	0.0066	0.022*	
C12	0.8874 (8)	−0.0179 (5)	0.1009 (3)	0.0179 (12)	
H12A	0.9794	0.0279	0.1305	0.021*	
H12B	0.8056	−0.0598	0.1268	0.021*	
C13	0.6595 (7)	−0.1495 (5)	−0.0815 (3)	0.0165 (12)	
H13A	0.6044	−0.0878	−0.1106	0.020*	
C14	0.6534 (7)	−0.2798 (5)	−0.1058 (3)	0.0161 (12)	
C15	0.6583 (7)	−0.3058 (5)	−0.1704 (3)	0.0185 (12)	
C16	0.6392 (8)	−0.4257 (6)	−0.1957 (3)	0.0210 (13)	
H16A	0.6420	−0.4397	−0.2400	0.025*	
C17	0.6158 (8)	−0.5253 (6)	−0.1554 (3)	0.0229 (13)	
H17A	0.6032	−0.6084	−0.1721	0.027*	
C18	0.6106 (8)	−0.5046 (6)	−0.0912 (3)	0.0210 (13)	
H18A	0.5949	−0.5729	−0.0635	0.025*	
C19	0.6288 (7)	−0.3818 (5)	−0.0674 (3)	0.0187 (12)	
H19A	0.6242	−0.3679	−0.0232	0.022*	
C20	1.0862 (8)	−0.1939 (6)	0.1114 (3)	0.0225 (13)	
H20A	1.1440	−0.2573	0.0877	0.034*	
H20B	1.1783	−0.1432	0.1375	0.034*	
H20C	1.0117	−0.2359	0.1393	0.034*	

### (2) 3,5-Bis[(E)-2-chlorobenzylidene]-1-methylpiperidin-4-one Atomic displacement parameters (Å^2^)

**Table:**

	*U*^11^	*U*^22^	*U*^33^	*U*^12^	*U*^13^	*U*^23^
Cl1	0.0287 (8)	0.0196 (7)	0.0305 (8)	−0.0023 (6)	0.0065 (6)	0.0026 (6)
Cl2	0.0292 (8)	0.0260 (8)	0.0176 (7)	−0.0003 (6)	0.0038 (6)	0.0015 (6)
O1	0.020 (2)	0.018 (2)	0.019 (2)	0.0055 (17)	0.0001 (17)	0.0049 (17)
N1	0.012 (2)	0.019 (3)	0.022 (2)	0.002 (2)	0.000 (2)	−0.002 (2)
C1	0.019 (3)	0.020 (3)	0.023 (3)	0.003 (2)	0.005 (2)	−0.002 (2)
C2	0.019 (3)	0.027 (3)	0.027 (3)	0.006 (3)	0.002 (3)	−0.002 (3)
C3	0.023 (3)	0.029 (4)	0.027 (3)	0.007 (3)	0.002 (3)	−0.012 (3)
C4	0.021 (3)	0.023 (3)	0.035 (4)	0.001 (3)	0.001 (3)	−0.005 (3)
C5	0.016 (3)	0.015 (3)	0.023 (3)	0.001 (2)	0.003 (2)	0.004 (2)
C6	0.012 (3)	0.018 (3)	0.023 (3)	0.003 (2)	0.004 (2)	−0.003 (2)
C7	0.014 (3)	0.019 (3)	0.023 (3)	0.001 (2)	0.005 (2)	0.004 (2)
C8	0.015 (3)	0.015 (3)	0.019 (3)	0.000 (2)	0.007 (2)	−0.002 (2)
C9	0.013 (3)	0.025 (3)	0.018 (3)	−0.004 (2)	0.007 (2)	0.000 (2)
C10	0.013 (3)	0.018 (3)	0.017 (3)	0.003 (2)	0.006 (2)	0.004 (2)
C11	0.020 (3)	0.017 (3)	0.019 (3)	0.000 (2)	0.003 (2)	−0.003 (2)
C12	0.019 (3)	0.017 (3)	0.018 (3)	0.000 (2)	0.004 (2)	−0.005 (2)
C13	0.016 (3)	0.019 (3)	0.014 (3)	0.000 (2)	0.002 (2)	0.000 (2)
C14	0.011 (3)	0.020 (3)	0.017 (3)	0.002 (2)	0.002 (2)	−0.001 (2)
C15	0.015 (3)	0.020 (3)	0.020 (3)	0.001 (2)	0.002 (2)	−0.002 (2)
C16	0.020 (3)	0.026 (3)	0.017 (3)	0.000 (3)	0.001 (2)	−0.005 (2)
C17	0.023 (3)	0.018 (3)	0.027 (3)	−0.004 (3)	0.001 (3)	−0.008 (3)
C18	0.018 (3)	0.024 (3)	0.020 (3)	−0.003 (3)	−0.001 (2)	−0.001 (2)
C19	0.016 (3)	0.021 (3)	0.019 (3)	0.001 (2)	0.001 (2)	−0.004 (2)
C20	0.025 (3)	0.020 (3)	0.022 (3)	0.002 (3)	0.001 (3)	0.001 (2)

### (2) 3,5-Bis[(E)-2-chlorobenzylidene]-1-methylpiperidin-4-one Geometric parameters (Å, º)

**Table:**

Cl1—C5	1.747 (6)	C10—C13	1.331 (8)
Cl2—C15	1.749 (6)	C10—C11	1.508 (8)
O1—C9	1.236 (7)	C11—H11A	0.9900
N1—C20	1.447 (7)	C11—H11B	0.9900
N1—C12	1.457 (7)	C12—H12A	0.9900
N1—C11	1.462 (7)	C12—H12B	0.9900
C1—C6	1.396 (8)	C13—C14	1.474 (8)
C1—C2	1.402 (8)	C13—H13A	0.9500
C1—H1A	0.9500	C14—C19	1.383 (8)
C2—C3	1.395 (9)	C14—C15	1.398 (8)
C2—H2A	0.9500	C15—C16	1.381 (8)
C3—C4	1.381 (9)	C16—C17	1.386 (8)
C3—H3A	0.9500	C16—H16A	0.9500
C4—C5	1.382 (8)	C17—C18	1.381 (8)
C4—H4A	0.9500	C17—H17A	0.9500
C5—C6	1.406 (8)	C18—C19	1.397 (8)
C6—C7	1.464 (8)	C18—H18A	0.9500
C7—C8	1.343 (8)	C19—H19A	0.9500
C7—H7A	0.9500	C20—H20A	0.9800
C8—C12	1.501 (8)	C20—H20B	0.9800
C8—C9	1.505 (8)	C20—H20C	0.9800
C9—C10	1.492 (8)		
			
C20—N1—C12	110.4 (4)	N1—C11—H11B	109.5
C20—N1—C11	110.1 (5)	C10—C11—H11B	109.5
C12—N1—C11	109.1 (4)	H11A—C11—H11B	108.1
C6—C1—C2	122.4 (6)	N1—C12—C8	112.1 (4)
C6—C1—H1A	118.8	N1—C12—H12A	109.2
C2—C1—H1A	118.8	C8—C12—H12A	109.2
C3—C2—C1	118.5 (6)	N1—C12—H12B	109.2
C3—C2—H2A	120.8	C8—C12—H12B	109.2
C1—C2—H2A	120.8	H12A—C12—H12B	107.9
C4—C3—C2	121.2 (6)	C10—C13—C14	128.4 (5)
C4—C3—H3A	119.4	C10—C13—H13A	115.8
C2—C3—H3A	119.4	C14—C13—H13A	115.8
C3—C4—C5	118.6 (6)	C19—C14—C15	116.3 (5)
C3—C4—H4A	120.7	C19—C14—C13	122.2 (5)
C5—C4—H4A	120.7	C15—C14—C13	121.4 (5)
C4—C5—C6	123.2 (6)	C16—C15—C14	122.9 (5)
C4—C5—Cl1	117.7 (5)	C16—C15—Cl2	118.0 (4)
C6—C5—Cl1	119.0 (5)	C14—C15—Cl2	119.1 (4)
C1—C6—C5	116.1 (5)	C15—C16—C17	118.8 (5)
C1—C6—C7	122.3 (5)	C15—C16—H16A	120.6
C5—C6—C7	121.5 (5)	C17—C16—H16A	120.6
C8—C7—C6	126.7 (5)	C18—C17—C16	120.4 (6)
C8—C7—H7A	116.6	C18—C17—H17A	119.8
C6—C7—H7A	116.6	C16—C17—H17A	119.8
C7—C8—C12	125.1 (5)	C17—C18—C19	119.1 (6)
C7—C8—C9	117.3 (5)	C17—C18—H18A	120.4
C12—C8—C9	117.6 (5)	C19—C18—H18A	120.4
O1—C9—C10	121.5 (5)	C14—C19—C18	122.4 (5)
O1—C9—C8	121.2 (5)	C14—C19—H19A	118.8
C10—C9—C8	117.2 (5)	C18—C19—H19A	118.8
C13—C10—C9	117.6 (5)	N1—C20—H20A	109.5
C13—C10—C11	124.0 (5)	N1—C20—H20B	109.5
C9—C10—C11	118.3 (5)	H20A—C20—H20B	109.5
N1—C11—C10	110.7 (5)	N1—C20—H20C	109.5
N1—C11—H11A	109.5	H20A—C20—H20C	109.5
C10—C11—H11A	109.5	H20B—C20—H20C	109.5
			
C6—C1—C2—C3	−0.4 (9)	C20—N1—C11—C10	−174.5 (4)
C1—C2—C3—C4	2.0 (9)	C12—N1—C11—C10	64.2 (6)
C2—C3—C4—C5	−1.6 (9)	C13—C10—C11—N1	149.3 (5)
C3—C4—C5—C6	−0.5 (9)	C9—C10—C11—N1	−33.2 (7)
C3—C4—C5—Cl1	180.0 (5)	C20—N1—C12—C8	175.3 (5)
C2—C1—C6—C5	−1.5 (8)	C11—N1—C12—C8	−63.6 (6)
C2—C1—C6—C7	−177.1 (5)	C7—C8—C12—N1	−149.2 (5)
C4—C5—C6—C1	1.9 (8)	C9—C8—C12—N1	31.0 (7)
Cl1—C5—C6—C1	−178.5 (4)	C9—C10—C13—C14	−172.8 (5)
C4—C5—C6—C7	177.6 (5)	C11—C10—C13—C14	4.8 (9)
Cl1—C5—C6—C7	−2.9 (7)	C10—C13—C14—C19	37.6 (9)
C1—C6—C7—C8	−39.9 (9)	C10—C13—C14—C15	−147.5 (6)
C5—C6—C7—C8	144.7 (6)	C19—C14—C15—C16	0.3 (8)
C6—C7—C8—C12	−5.9 (9)	C13—C14—C15—C16	−175.0 (5)
C6—C7—C8—C9	173.9 (5)	C19—C14—C15—Cl2	−179.0 (4)
C7—C8—C9—O1	−3.8 (8)	C13—C14—C15—Cl2	5.8 (7)
C12—C8—C9—O1	176.1 (5)	C14—C15—C16—C17	−0.6 (9)
C7—C8—C9—C10	179.6 (5)	Cl2—C15—C16—C17	178.6 (4)
C12—C8—C9—C10	−0.6 (7)	C15—C16—C17—C18	0.4 (9)
O1—C9—C10—C13	2.9 (8)	C16—C17—C18—C19	0.2 (9)
C8—C9—C10—C13	179.6 (5)	C15—C14—C19—C18	0.3 (8)
O1—C9—C10—C11	−174.7 (5)	C13—C14—C19—C18	175.5 (5)
C8—C9—C10—C11	1.9 (7)	C17—C18—C19—C14	−0.5 (9)

### (2) 3,5-Bis[(E)-2-chlorobenzylidene]-1-methylpiperidin-4-one Hydrogen-bond geometry (Å, º)

**Table:**

*D*—H···*A*	*D*—H	H···*A*	*D*···*A*	*D*—H···*A*
C4—H4*A*···Cl2^i^	0.95	2.85	3.587 (7)	135

Symmetry code: (i) *x*+1/2, −*y*+1/2, *z*+1/2.
